# Autoimmune Encephalitis Associated With Anti-Contactin-Associated Protein-Like 2 (Anti-CASPR2) Antibodies in an Elderly Patient: A Case Report

**DOI:** 10.7759/cureus.102203

**Published:** 2026-01-24

**Authors:** Raúl Anwar Garcia-Santos, L. Jimena Gómez-Rodríguez, M. Fernanda Mercado-Torres, Miranda H Urquijo-Arteaga

**Affiliations:** 1 Neurology, National Institute of Neurology and Neurosurgery, Mexico City, MEX; 2 Internal Medicine, Hospital Médica Sur, Mexico City, MEX; 3 Internal Medicine, Hospital Medica Sur, Mexico City, MEX; 4 Intensive Care Unit, Star Médica Hospital Querétaro, Santiago de Querétaro, MEX

**Keywords:** anti-caspr2, autoimmune encephalitis, elderly, focal status epilepticus, immunotherapy

## Abstract

The leading cause of epilepsy in the elderly is cerebrovascular disease. Autoimmune encephalitis (AE) is an increasingly recognized cause of new-onset seizures in older adults, particularly when no structural lesion or infectious aetiology is identified and when there are unexplained new-onset seizures, particularly in focal status epilepticus. We report the case of a 77-year-old male with anti-contactin-associated protein-like 2 (anti-CASPR2) encephalitis whose initial brain MRI and routine labs were unremarkable. Despite the negative imaging and routine blood tests, clinical evaluation yielded a high Antibody Prevalence in Epilepsy and Encephalopathy (APE2) and the Response to Immunotherapy in Epilepsy 2 (RITE2) scores (8 points), justifying the early initiation of methylprednisolone and intravenous immunoglobulin. Following intensive management and antibody confirmation in serum and cerebrospinal fluid tests, the patient achieved significant clinical improvement, seizure control, and successful tapering of antiseizure medications. This case emphasizes that AE is an important and potentially treatable cause of new-onset epilepsy in the elderly. Early recognition using validated clinical scales such as APE2 and RITE2 can support prompt immunotherapy and improve outcomes beyond antiseizure treatment alone.

## Introduction

Late-onset epilepsy has a substantial impact on the quality of life of older adults, and it increases healthcare costs for society. The annual incidence has been estimated at 85 cases per 100,000 individuals between 65 and 69 years of age, and 159 per 100,000 individuals over 80 years [1]. Approximately 25% of new-onset epilepsy occurs in the elderly, and in about 50% of these cases, an underlying etiology can be identified [1].

Among new-onset epilepsy etiologies in the elderly are cerebrovascular disease (30-50% of cases), including both ischemic and hemorrhagic events, as well as primary neurodegenerative diseases such as Alzheimer's disease (approximately 10-20% of the cases) [1].

Autoimmune encephalitis (AE) is considered one of the most frequent causes of non-infectious acute encephalitis; it is estimated that up to 20% of encephalitis cases in Northern Europe are immune-mediated [2]. Annual incidence of all types of encephalitis worldwide is reported to be five to eight cases per 100,000 individuals; however, in up to 50% of the cases, no specific etiology can be identified [3]. The exact incidence of AE remains unknown; nevertheless, during the last decade, there has been an increase in reported cases of this condition [3].

AE is characterized by an acute or subacute onset that can become chronic. Recently, the emerging concept of “epilepsy associated with AE” has been proposed, in which inflammation leads to a structural lesion that becomes the main contributor to seizure predisposition over time [4]. Suggested etiologies that can trigger AE include tumors (paraneoplastic syndromes), infections, or may remain cryptogenic. The first category is strongly associated with T-cell responses that target neurons. There is another category that includes autoantibodies against extracellular epitopes of channels, receptors, and other proteins, such as the anti-N-methyl-D-aspartate receptor [2]. Also, there are diseases that do not fit neatly into either of these defined categories, those characterized by autoantibodies to intracellular synaptic proteins, such as antibodies against glutamic acid decarboxylase 65 (GAD 65) [5].

Anti-contactin-associated protein-like 2 (CASPR2) autoimmune disease presents as a spectrum of neurological syndromes resulting from autoantibodies directed against the CASPR2 protein. CASPR2 is a transmembrane axonal protein that functions in organizing and concentrating voltage-gated potassium channels (VGKCs) at the juxtaparanodes of myelinated axons. It is widely expressed both in the central nervous system (CNS) and the peripheral nervous system (PNS) [5].

The most common presentations in AE associated with anti-CASPR2 include limbic encephalitis, Morvan syndrome, and peripheral nerve hyperexcitability syndromes. Limbic encephalitis manifests with cognitive impairment, seizures, and psychiatric symptoms, while Morvan syndrome is defined by a combination of encephalopathy, neuromyotonia, dysautonomia, and agrypnia excitata (severe insomnia, dream-like stupor, sympathetic hyperactivity, and motor agitation) [5,6]. Peripheral nerve hyperexcitability may present as neuromyotonia, fasciculations, and neuropathic pain [6]. Also, the most commonly associated malignancy is thymoma [7].

## Case presentation

A 77-year-old male patient, previously healthy, presented with seizures one month prior to hospitalization. Seizure semiology consisted of an unusual pharyngeal sensation (referred to as “throat strangeness”), moderate occipital-to-holocranial headache (intensity 5/10), dysphasia, disconnection from the environment, behavior arrest, clonic movements in the superior lips, left superior limb, occasionally involving the left inferior limb, incoherent speech, and rarely, generalized tonic-clonic seizures.

Treatment with maximum doses of lacosamide (LCS) (200 mg BID) was initiated without seizure freedom. Levetiracetam (LEV) (2 grams BID) was added until seizure control was achieved. The patient continued to have headaches daily with medium intensity. He came to the emergency department due to uncontrolled seizures. Two days preceding hospitalization, he had consumed alcohol and had forgotten to take his medication. He had presented multiple seizures with increasing frequency, described by family members as incoherent speech (confusing conversations and speaking without meaning).

**Figure 1:**
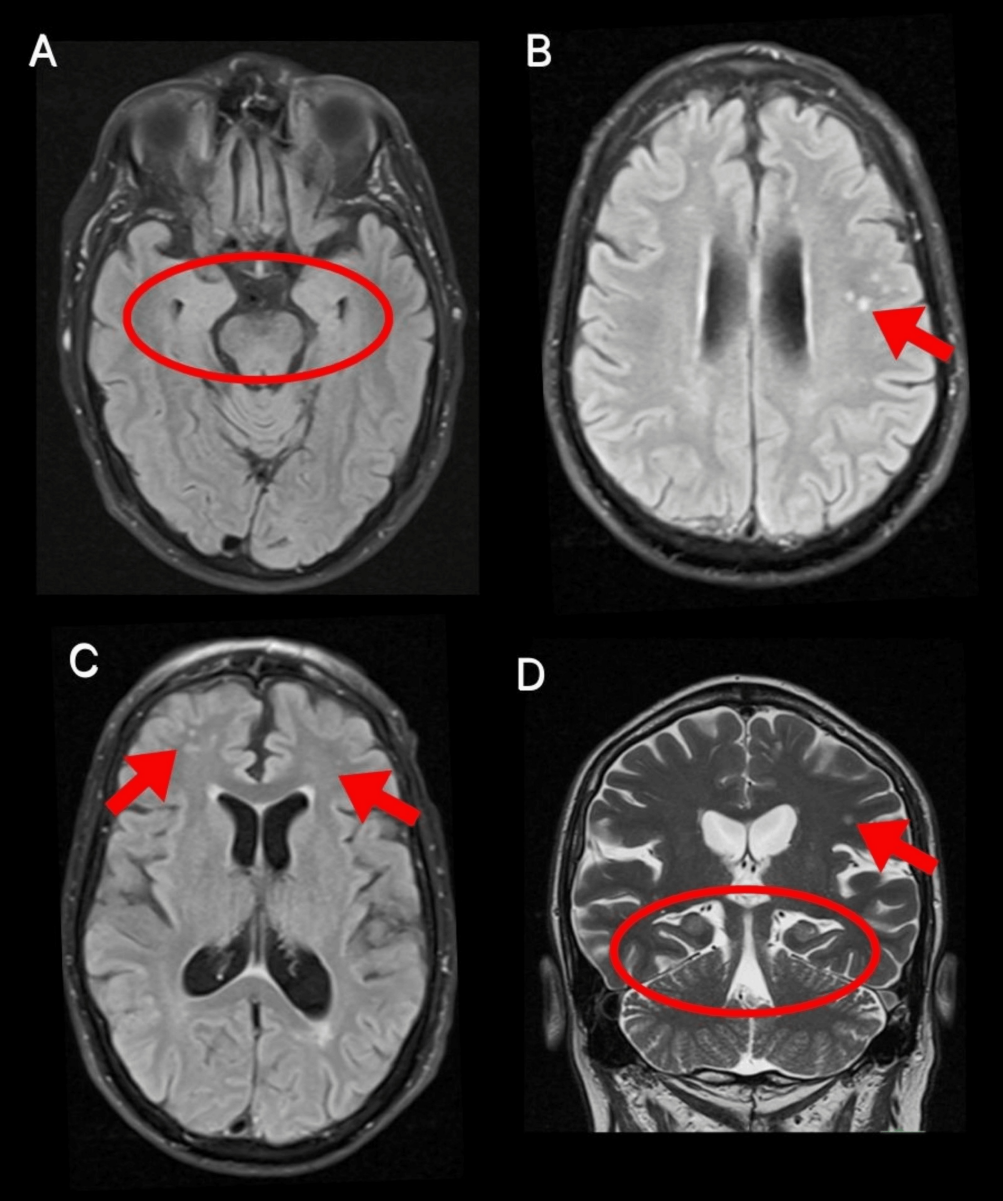
Brain MRI (A) FLAIR sequence, coronal view: hippocampi (red circle) appear symmetric with no signal abnormalities. (B) FLAIR sequence, axial view: multiple punctate subcortical hyperintensities in the left parietal region (red arrow). (C) FLAIR sequence, axial view: multiple punctate hyperintensities in the right frontal and periventricular regions (red arrows). (D) T2-weighted sequence, coronal view: nodular hyperintensity in the left parietal region (red arrow) with symmetric hippocampi (red circle). FLAIR: fluid-attenuated inversion recovery

**Figure 2:**
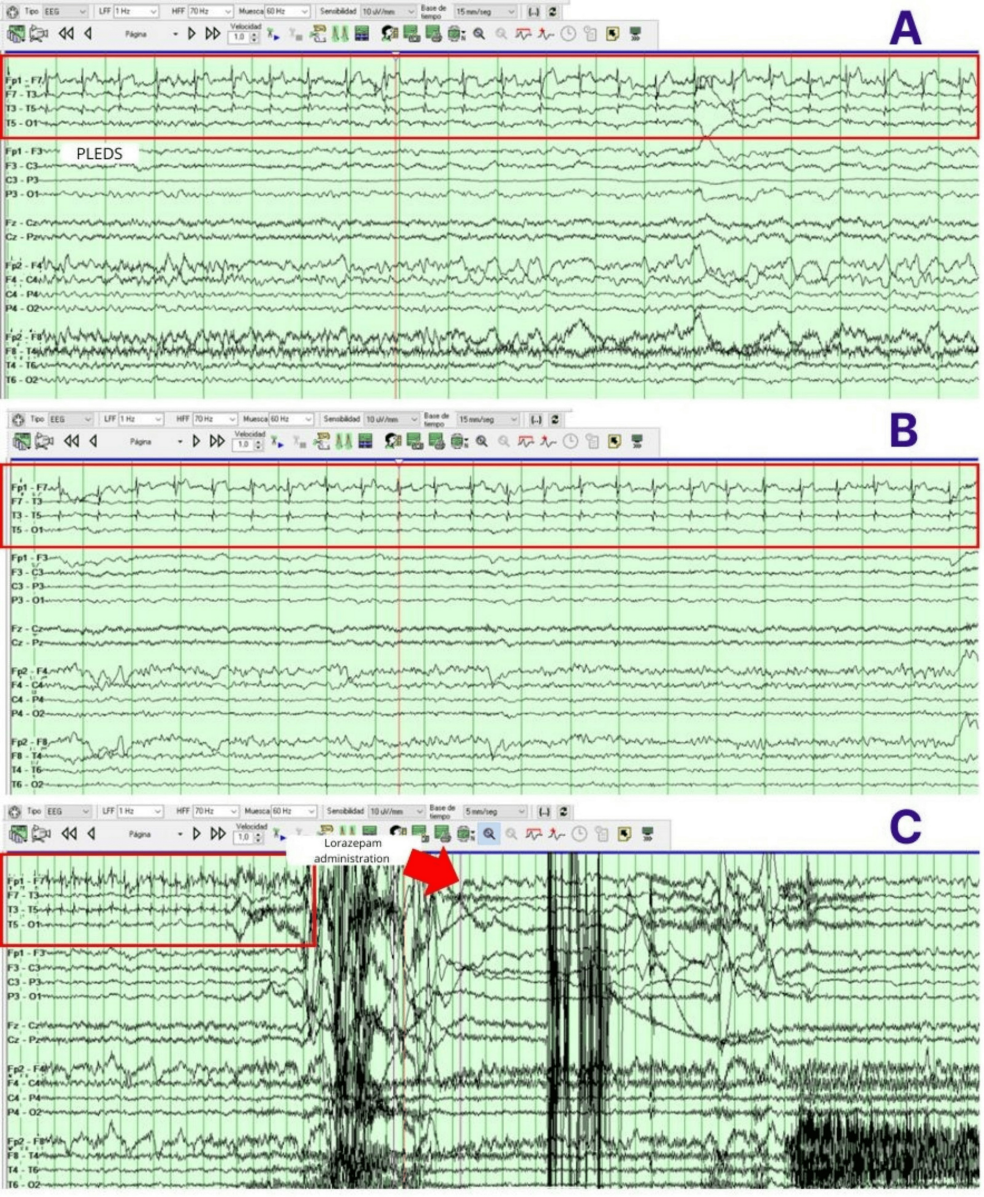
EEG Bipolar longitudinal “double-banana” montage showing: (A-B) Periodic lateralized epileptiform discharges (PLEDs) in the temporal and frontal regions (F7-T3-T5-O1) (red rectangles), clinically associated with dysarthria and clonic movements of the upper extremities. (C) PLEDs with subsequent suppression following administration of lorazepam (red arrow).

**Figure 3:**
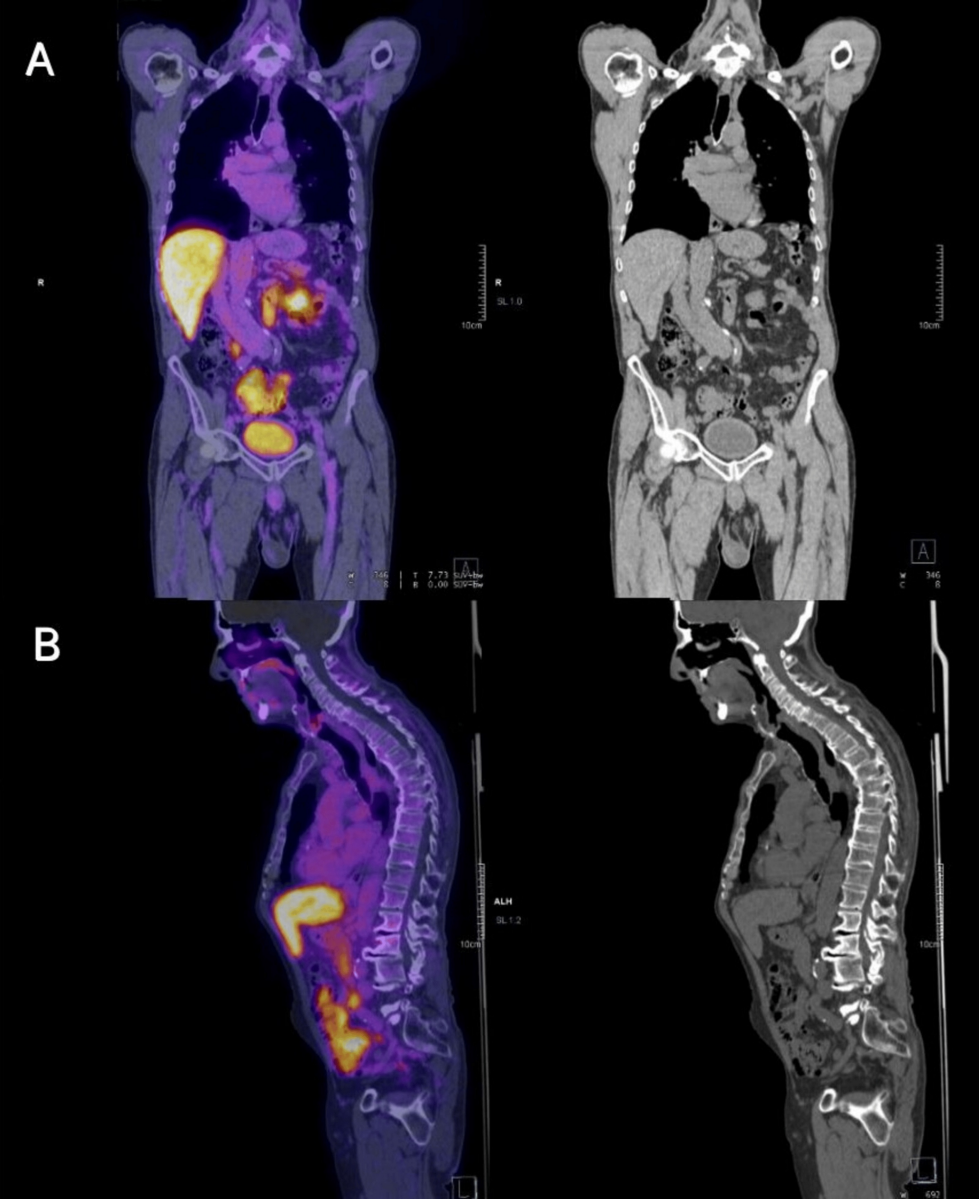
18-fluorodesoxyglucose positron emission tomography-computed tomography (18 FDG PET-CT) (A) Coronal view without evidence of increased anormal metabolism compatible with neoplastic activity. (B) Sagittal view without evidence of increased anormal metabolism compatible with neoplastic activity.

During his hospitalization, management was initiated with lorazepam 8 mg IV, LEV 60 mg/kg, and LCS 400 mg IV. Routine blood tests showed hyperglycemia and mild hyponatremia; the rest were unremarkable (Table 1). A brain MRI with gadolinium was requested, which showed an area of malacia in the left cerebellar hemisphere, microvascular disease, Fazekas 2 (multiple punctate subcortical hyperintensities in the left parietal and frontal periventricular region), and decreased cortico-subcortical volume (Figure 1). EEG revealed mild diffuse dysfunction, as well as some increase in bilateral frontotemporal excitability with left predominance. A lumbar puncture was performed with an opening pressure of 80 mmH_2_O, and mildly elevated protein concentration (0 cells, proteins 85, glucose 96) (Table 2). An autoimmune panel in serum and cerebrospinal fluid (CSF) was requested, identifying CASPR2 IgG antibody at a 1:10 dilution, confirmed at 1:100 dilution. Due to the presentation of non-convulsive status epilepticus, the patient required advanced airway management and continuous midazolam sedation for 48 hours, with ongoing EEG monitoring in the intensive care unit (Figure 2). During his course, methylprednisolone 1 gram intravenously every 24 hours for five days was administered, and intravenous immunoglobulin at 0.4/kg/day for five days was initiated, along with LCS 200 mg every 12 hours and valproic acid 600 mg every 12 hours. A screening protocol for neoplasms was performed with PET and whole-body CT, which did not reveal any malignancy (Figure 3). The patient’s clinical course was favorable, with a reduction in seizures, and the doses of antiepileptic drugs were gradually tapered.

**Table 1:** Blood tests The patient's blood test showing hyperglycemia and mild hyponatremia; the rest are within the reference range.

Blood test items	Abbreviation	Patient's results	Units	Reference interval
Hemoglobin	Hb	15.5	g/dL	12.6-16.1
Hematocrit	Hct	47.0	%	36.8-47.3
Mean corpuscular volume	MCV	94.1	fL	82.3-96.3
Red blood cells	RBC	5.0	x10^6/μL	4.03-5.28
Platelets	PLT	239	x10^3/μL	160-441
White blood cells	WBC	7.3	x10^3/μL	3.59-9.70
Glucose	Glu	297	mg/dL	72-99
Blood urea nitrogen	BUN	18.6	mg/dL	9.0-21.0
Creatinine	Cr	1.0	mg/dL	0.60-1.03
Sodium	Na	135	mmol/L	138-145
Potassium	K	4.8	mmol/L	3.9-5.3
Chloride	Cl	103	mmol/L	103-110

**Table 2:** Patient's cerebrospinal fluid (CSF) test CSF analysis showing elevated opening pressure, increased protein concentration (proteinorrachia), and elevated CSF glucose levels.

CSF test items	Patient's results	Units	Reference interval
Color	Colorless	-	Colorless
Aspect	Transparent	-	Transparent
White blood cells	0	WBC/μL	0
Red blood cells	0	RBC/μL	0
CSF glucose	96	mg/dL	40-70
CSF total proteins	85	mg/dL	15-45
Opening pressure	80	mmH_2_O	7-18

During the evaluation of the case, the Antibody Prevalence in Epilepsy and Encephalopathy (APE2) score was calculated, yielding a total of 8 points, based on the following criteria: refractoriness to two antiseizure medications, neuropsychiatric manifestations, facial dyskinesias, CSF abnormalities, and disease duration. Similarly, the Response to Immunotherapy in Epilepsy 2 (RITE2) score was 8 points, considering the duration of symptoms, neuropsychiatric changes, facial dyskinesias, treatment refractoriness, CSF findings, and the presence of CASPR2 antibodies.

## Discussion

AE in the elderly is characterized by a subacute onset of neuropsychiatric symptoms, cognitive impairment, confusion, behavioral changes, psychosis, seizures (including faciobrachial dystonic seizures), and movement disorders [2]. However, these symptoms often overlap with other neuropathologies such as stroke, dementia, or viral encephalitis, making the diagnosis of AE particularly challenging. This diagnostic uncertainty is well recognized in the literature, where the broad and nonspecific presentation of AE often leads to misclassification or delayed recognition [8].

In older adults, new-onset seizures warrant special attention, as this age group has the highest incidence of epilepsy across the lifespan. Current epidemiological data show that individuals over 60 have a sharply increased risk of both acute symptomatic seizures and epilepsy, with incidence rates exceeding 100 per 100 000 persons and rising progressively with age [1]. Unlike younger populations, where idiopathic or genetic epilepsies are more common, seizures in the elderly are predominantly focal-onset and symptomatic in origin, most often resulting from cerebrovascular disease, neurodegeneration, brain tumors, trauma, or immune-mediated mechanisms [1]. Focal seizures, particularly those with impaired awareness, or focal status epilepticus, are the most frequent presentations, and generalized seizures are comparatively less common. These epidemiologic patterns are clinically relevant, as they mirror the seizure semiology of AE, especially in disorders associated with CASPR2 and LGI1 autoantibodies.

The most common subtypes of AE in the elderly are associated with anti-LG11, anti-CASPR2, and anti-IgLON5 antibodies [9]. The latter typically manifests clinically with focal seizures, including focal status epilepticus, in contrast to anti-NMDA receptor encephalitis, which more often presents with generalized seizures [4]. In this context, our patient’s focal status epilepticus, refractory to treatment, was consistent with CASPR2 antibody-associated encephalitis.

To isolate the autoimmune etiology from the broad presentation of late-onset epilepsy, a systematic differential diagnosis was performed to exclude more common conditions. Although cerebrovascular disease accounts for up to 50% of new epilepsy diagnoses in the elderly [1], MRI in this patient showed only chronic small-vessel changes (Fazekas 2) without acute ischemia, hemorrhage, or cortical lesions. Furthermore, the patient had no acute focal deficits, and the seizure semiology, characterized by recurrent dysphasia, behavioral arrest, and faciobrachial-like involvement, was not typical of post-stroke epilepsy. Neurodegenerative disorders such as Alzheimer’s disease, which also contribute significantly to late-onset epilepsy, particularly with focal impaired-awareness seizures [1], were ruled out due to the lack of preceding cognitive decline or functional impairment, and the absence of hippocampal atrophy on neuroimaging. Infectious encephalitis was deemed unlikely given the absence of fever, systemic symptoms, or CSF pleocytosis. The CSF profile instead showed isolated protein elevation, a pattern more characteristic of autoimmune than infectious processes [10]. Metabolic and toxic triggers, including electrolyte imbalances, hypoglycemia, and uremia, were excluded via normal laboratory parameters. While alcohol intake likely precipitated breakthrough events, it did not account for the one-month history of recurrent seizures. Finally, PET-CT ruled out underlying malignancy, including thymoma, which can be associated with anti-CASPR2 autoimmunity [8], and no structural lesions were identified that could justify a new epileptogenic focus. Following the exclusion of these etiologies, the presence of CASPR2 antibodies in both serum and CSF, combined with high APE2 and RITE2 scores, confirmed the autoimmune mechanism.

CASPR2 encephalitis frequently presents with focal seizures or focal status epilepticus, often with normal or nonspecific MRI and EEG findings early in the disease course [8]. These features aligned closely with the patient’s presentation, further reinforcing the diagnosis. Thus, early immunotherapy was justified and contributed to the favorable clinical outcome.

Given the limitations of neuroimaging and EEG, both of which may appear normal early in the disease course, clinical scoring systems provide valuable guidance in raising suspicion for AE and prompting timely testing and treatment [8]. The APE2 (Table 3) and RITE2 (Table 4) scores were particularly informative in this case: the patient scored 8 on APE2 and 8 on RITE2, indicating a high likelihood of autoimmune etiology and supporting the early initiation of immunotherapy. These tools have demonstrated high sensitivity for predicting antibody positivity and seizure responsiveness to immunotherapy [10,11].

**Table 3:** Components of the APE2 score This table summarizes all criteria considered in the APE2 scoring system. APE2: Antibody Prevalence in Epilepsy and Encephalopathy; FLAIR: fluid-attenuated inversion recovery; CSF: cerebrospinal fluid

Clinical feature	Value (total max = 18)
New onset, rapidly progressive mental status changes that developed over 1-6 weeks or new onset seizure activity (within one year of evaluation)	+1
Neuropsychiatric changes; agitation, aggressiveness, emotional lability	+1
Autonomic dysfunction [sustained atrial tachycardia or bradycardia, orthostatic hypotension (≥20 mmHg fall in systolic pressure or ≥ 10 mmHg fall in diastolic pressure within three minutes of quiet standing), hyperhidrosis, persistently labile blood pressure, ventricular tachycardia, cardiac asystole or gastrointestinal dysmotility	+1
Viral prodrome (rhinorrhea, sore throat, low grade fever) to be scored in the absence of underlying systemic	+2
Faciobrachial dystonic seizures	+3
Facial dyskinesias, to be scored in the absence of faciobrachial dystonic seizures	+2
Seizure refractory to at least two anti-seizure medications	+2
CSF findings consistent with inflammation (elevated CSF protein >50 mg/dL and/or lymphocytic pleocytosis > 5 cells/mcL, if the total number of CSF RBC is < 1000 cells/mcL)	+2
Brain MRI suggesting encephalitis (T2/FLAIR hyperintensity restricted to one or both medial temporal lobes, or multifocal in grey matter, white matter, or both compatible with demyelination or inflammation)	+2
Systemic cancer diagnosed within 5 years of neurological symptom onset (excluding cutaneous squamous cell carcinoma, basal cell carcinoma, brain tumor, cancer with brain metastasis)	+2

**Table 4:** Components of the RITE2 score RITE2: Response to Immunotherapy in Epilepsy 2; FLAIR: fluid-attenuated inversion recovery; CSF: cerebrospinal fluid

Clinical feature	Value (total max = 22)
New onset, rapidly progressive mental status changes that developed over 1-6 weeks or new onset seizure activity (within one year of evaluation)	+1
Neuropsychiatric changes; agitation, aggressiveness, emotional lability	+1
Autonomic dysfunction [sustained atrial tachycardia or bradycardia, orthostatic hypotension (≥20 mmHg fall in systolic pressure or ≥ 10 mmHg fall in diastolic pressure within three minutes of quiet standing), hyperhidrosis, persistently labile blood pressure, ventricular tachycardia, cardiac asystole or gastrointestinal dysmotility	+1
Viral prodrome (rhinorrhea, sore throat, low grade fever) to be scored in the absence of underlying systemic	+2
Faciobrachial dystonic seizures	+3
Facial dyskinesias, to be scored in the absence of faciobrachial dystonic seizures	+2
Seizure refractory to at least two anti-seizure medications	+2
CSF findings consistent with inflammation (elevated CSF protein >50 mg/dL and/or lymphocytic pleocytosis > 5 cells/µL, if the total number of CSF RBC is < 1000 cells/µL)	+2
Brain MRI suggesting encephalitis (T2/FLAIR hyperintensity restricted to one or both medial temporal lobes, or multifocal in grey matter, white matter, or both compatible with demyelination or inflammation)	+2
Systemic cancer diagnosed within 5 years of neurological symptom onset (excluding cutaneous squamous cell carcinoma, basal cell carcinoma, brain tumor, cancer with brain metastasis)	+2
Immunotherapy initiated within 6 months of symptom onset	+2
Neural plasma membrane autoantibody detected (NMDAR, GABAAR, GABABR, AMPAR, DPPX, mGluR1, mGluR2, mGluR5, LGI1, IgLON5, CASPR2 or MOG)	+2

Most patients with AE experience only acute symptomatic seizures during the active phase of the disease, which typically resolve with appropriate immunotherapy. Therefore, early evaluation and treatment are essential - even in the absence of serological confirmation - when clinical suspicion is high. Only a small proportion (less than 15%) go on to develop chronic epilepsy, as the underlying process is predominantly inflammatory and reversible in most cases [12].

Early AE treatment with immunotherapy can reduce seizure frequency or facilitate seizure termination, and is associated with improved long-term outcomes, including preservation of cognitive function [4,10]. In this case, immunotherapy with intravenous methylprednisolone followed by intravenous immunoglobulin, combined with antiseizure polytherapy, was associated with a favorable outcome and seizure reduction. This outcome is consistent with existing evidence showing that seizures in AE are poorly responsive to antiseizure medications alone (with a control rate of ~10%), whereas immunotherapy significantly increases the likelihood of clinical improvement and seizure control [4].

## Conclusions

In elderly patients with new-onset epilepsy and unremarkable initial studies, an autoimmune etiology should be considered, particularly in the presence of focal status epilepticus. The APE2 and RITE2 scores are valuable clinical tools that aid in the identification and management of patients with seizures of possible autoimmune origin, especially in the context of AE. Their application facilitates early initiation of immunotherapy, which is crucial for improving long-term seizure control and functional outcomes.
